# Prevalence and patterns of illicit drug use in people with human immunodeficiency virus infection in Korea

**DOI:** 10.1371/journal.pone.0249361

**Published:** 2021-04-01

**Authors:** Soon Ok Lee, Jeong Eun Lee, Shinwon Lee, Sun Hee Lee, Jin Suk Kang

**Affiliations:** 1 Department of Internal Medicine, Pusan National University School of Medicine, Medical Research Institute, Pusan National University Hospital, Busan, Korea; 2 Division of Infectious Disease, Department of Internal Medicine, Inje University College of Medicine, Inje University Busan Paik Hospital, Busan, Korea; University of Cincinnati College of Medicine, UNITED STATES

## Abstract

**Background:**

Data on illicit drug use by Korean people infected with HIV are lacking. Here, we examined the prevalence and patterns of illicit drug use among people infected with HIV in Korea.

**Material and methods:**

In this cross-sectional study, we included all patients infected with HIV who visited a tertiary care hospital in Korea from January 1990 to May 2020. Sociodemographic data of patients, including drug use, were collected at the first visit and during follow-up.

**Results:**

Among 1,267 patients, 5.13% reported the use of an illicit drug in their lifetime, and 2.61% were users of injection drugs. The most commonly used drugs were cannabis/marijuana and methamphetamine, followed by nitrite inhalants, cocaine, dextromethorphan, carisoprodol, and 3,4-methylenedioxymethamphetamine. The illicit drug users tended to be younger than non-users, homosexuals/bisexuals, and infected with hepatitis C virus (HCV); they had a higher CD4^+^ cell count than non-users. Among 65 users of illicit drugs, only 24.6% revealed their drug use at the first visit interview, and 24.6% reported using two or more drugs in their lifetime. Individuals who used injection drugs were more likely to be arrested for drug-related charges than the users of non-injection drugs. Moreover, they tended to be heavy users of illicit drugs, to report using two or more drugs in their lifetime, and to be HCV seropositive. In contrast, the users of non-injection drugs were more likely to be experimental users and to start using drugs overseas for the first time, but less likely to report their drug use at the first interview.

**Conclusions:**

The prevalence of illicit drug use in people with HIV infection in Korea may have been underestimated. Further research based on more accurate measurements are warranted.

## Introduction

The use of illicit drugs has become a major cause of the transmission of human immunodeficiency virus (HIV) in several parts of the world [1]. HIV can be transmitted either directly through the sharing of needles or other equipment or indirectly due to impaired judgment, which leads to unprotected sex with an infected person. The prevalence and patterns of illicit drug use among people with HIV may substantially vary across countries and over time, depending on a combination of factors, including socioeconomic circumstances, government policies, criminal justice system, and systems for the reduction and prevention of negative effects.

In Korea, the use of illicit drugs has increased since the mid-1990s. The annual number of arrests for drug offenses in Korea sharply increased from 2,968 in 1992 to 10,304 in 1999, and since then the number has fluctuated between 8,000 and 14,000 per year until recently [2]. This increase is likely due to the rapid globalization after relaxation of the travel restriction policy by Korea in 1989 and increased use of the internet and social networking services, which make it easier to procure drugs [3,4]. Methamphetamine accounts for most drug-related arrests, followed by cannabis [2,5]. In a web-based survey, inhalants were found to be the most commonly used drug class among adolescents [4]. Recently, the use of new psychoactive substances including synthetic cannabinoids and phenethylamines has been increasingly identified in Korea [5].

However, little is known about the prevalence of illicit drug use among people with HIV infection in Korea. The use of injection drugs has been considered to play a minor role in the HIV epidemic in Korea. According to the HIV surveillance data from the Korean Centers for Disease Control and Prevention (KCDC), the users of injection drugs accounted for 0.04% of the 12,320 individuals diagnosed with HIV from 1985 to 2017 [6]. Furthermore, a recent HIV cohort study showed that the users of injection drugs in Korea accounted for only 0.07% of the 1,442 enrolled patients [7]. In a previous seroprevalence study in 318 users of injection drugs arrested between 2007 and 2010 in Korea, 48.4% of the patients were infected with hepatitis C virus (HCV), but there was no case of HIV infection [8].

In contrast to the abovementioned results, we previously reported that users of injection drugs accounted for 1.8% of 790 patients with HIV infection who were tested for HCV serology [9]. In a subsequent HCV serologic test, seroconversion was observed in three users of injection drugs, and this suggested that the prevalence of injection drug use among people with HIV infection in Korea might have been underestimated [9]. Moreover, so far, there have been no attempts to better understand the situation of illicit drug use among people with HIV infection in Korea.

To address this gap, we examined the prevalence and patterns of illicit drug use among people with HIV infection in Korea. Furthermore, we compared the epidemiological characteristics between users of injection and non-injection drugs.

## Materials and methods

### Study population

In this cross-sectional study, we included all patients with HIV infection who visited Pusan National University Hospital, Busan, Korea, from January 1990 to May 2020. Patients aged <15 years at the time of enrollment and ethnic non-Koreans were excluded. Patients who were lost to follow-up after visiting the study hospital for treatment other than HIV care were also excluded.

The medical records of the patients were reviewed to obtain epidemiological and clinical data. Socio-demographic data, including the history of drug use, were collected at the first visit as a part of the clinical interview. Because interviews at the first visit could potentially underestimate the prevalence of illicit drug use, we also obtained the history of illicit drug use and sexual contact as a part of the routine care practice during follow-up. Patients who reported the use of illicit drugs were enquired about the drug use characteristics, including the type of drug or name of street, number of drugs used in lifetime, frequency and timing of drug use, effect of drugs on their sexual behavior, countries where they used the drug, whether they had been arrested or imprisoned, and experience of accessing the support programs. If the name of the illicit drug used was not specified, it was classified as undescribed. Additionally, the laboratory data of CD4 cell count, HIV-1 viral load, hepatitis C antibody, hepatitis B antibody, and syphilis serology were collected.

The illicit drug was defined as a substance that is legally prohibited in Korea under the Act on the Control of Narcotics; it included narcotics, psychotropic drugs or substances, cannabis, and some substances designated as temporary narcotics, such as nitrite inhalants [10]. We also included the use of other substances such as sniffing of glue prohibited by other laws, including the Act on the Protection of Children and Youth against Sex Offenses and Chemical Substance Control Act, in Korea. Drug use for therapeutic purposes was excluded from the analysis. The use of injection drugs was defined as the administration of substances by piercing the skin with a needle and a syringe. The use of non-injection drugs was defined as the administration of substances via oral ingestion or nasal inhalation. A drug that was used in both injection and non-injection forms was classified as an injection drug. The consumption levels of all substances were classified as follows: experimental use (1–2 times in one’s life), occasional use (3–9 times in one’s life), and heavier use (10 or more times in one’s life) [11,12].

The study protocol was approved by the Institutional Review Board of Pusan National University Hospital (IRB number: 2006-008-091). The Institutional Review Board waived the need for informed consent.

### Statistical analyses

Statistical analyses were performed using SPSS version 22.0 (IBM SPSS Statistics, USA). Categorical variables were compared using Pearson’s chi-squared test or Fisher’s exact test, and non-categorical variables were tested using Student’s *t*-test or Mann–Whitney *U*-test. All tests of significance were two-tailed. Results with a P value of <0.05 were considered significant.

## Results

Totally, 1,341 people with HIV infection visited the study hospital during the study period. Among them, 43 ethnic non-Koreans, 2 patients aged <15 years, and 29 patients whose first visit was not for HIV care and were subsequently lost to follow-up were excluded. Finally, 1,267 patients were enrolled for analysis. The mean age of patients was 42.1 ± 12.4 years (mean ± 95% standard deviation), and 89.3% of the patients were men (Table 1). Among the 1,267 patients, 65 (5.13%) reported the use of an illicit drug in their lifetime and 33 (2.61%) were users of injection drugs.

**Table 1:** Baseline characteristics of HIV-infected patients analyzed by comparing illicit drug users and non-users.

Variable	Total (n = 1,267)	Users of illicit drugs (n = 65)	Non-users of illicit drugs (n = 1,202)	P value
Sex				0.105^a^
Male	1,132 (89.3)	62(95.4)	1,070 (89)	
Female	135 (10.7)	3 (4.6)	132 (11)	
Age at presentation, years	42.1 ± 12.4	39.3 ± 10.6	42.3 ± 12.5	0.031^c^
Marriage				0.051^a^
Unmarried	643 (50.7)	42 (64.6)	601(50)	
Ever married	601 (47.4)	23 (35.4)	578 (48.1)	
Unrecorded	23 (1.8)	0 (0)	23 (1.9)	
Sexual behavior				<0.001^a^
Heterosexual	643 (50.7)	18 (27.7)	625 (52.0)	
Homo/bisexual	577 (45.5)	47 (72.3)	530 (44.1)	
Unrecorded	47 (3.7)	0 (0)	47 (3.9)	
Year of initial presentation to HIV care				0.856 ^a^
1990–2000	177 (14.0)	9 (13.8)	168 (14.0)	
2001–2010	603 (47.6)	33 (50.8)	570 (47.4)	
2011–2020	487 (38.4)	23 (35.4)	464 (38.6)	
Follow-up period, years	6.22 ± 6.22	7.07 ± 6.12	6.17 ± 6.23	0.258^c^
CD4^+^ cell count, cells/mm^3^	272.6 ± 236.3	358.7 ± 236.4	267.8 ± 235.5	0.003^c^
HBV serology positive	99/1,159 (8.5)	6/60 (10.0)	93/1,099 (8.5)	0.678^a^
HCV serology positive	54/1,107 (4.9)	14/59 (23.7)	40/1,048 (3.8)	<0.001^b^
Syphilis serology positive	428/1,125 (38)	28/61 (45.9)	400/1,064 (37.6)	0.194 ^a^

^a^ Chi-squared test.

^b^ Fisher’s exact test.

^c^ Student’s *t*-test.

Values are presented as mean ± SD, number (%).

HIV, human immunodeficiency virus; HBV, hepatitis B virus; HCV, hepatitis C virus.

There were differences in several demographic features between illicit drug users and non-users (Table 1). Illicit drug users were younger than non-users (39.3 vs. 42.3 years, p = 0.031) and were more likely to be homosexuals/bisexuals (72.3% vs. 44.1%, p < 0.001). The HCV seropositive rate was significantly higher in illicit drug users than in non-users (23.7% vs. 3.8%, p < 0.001). The CD4 cell count at presentation to the hospital was higher in the illicit drug users than in non-users (358.7 ± 236.4/μL vs. 267.8 ± 235.5/μL, p = 0.003).

**Fig 1:**
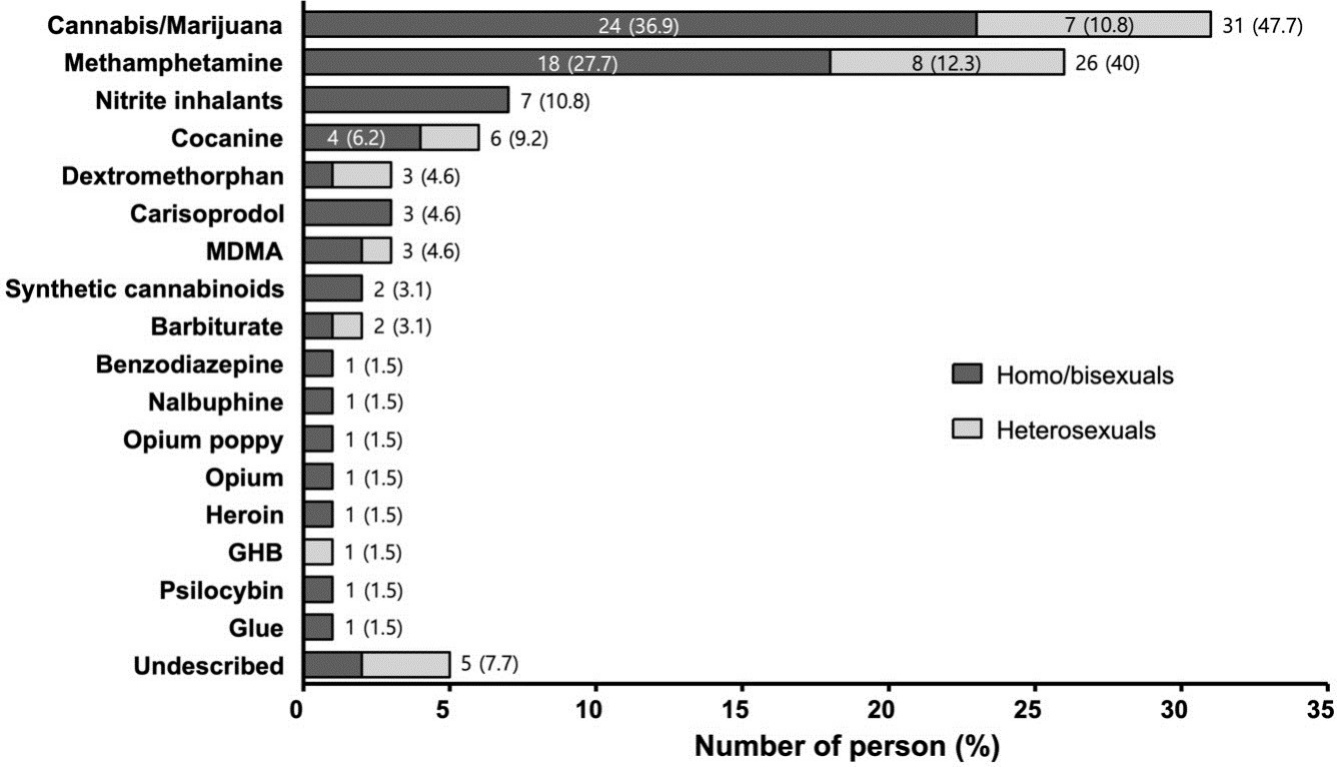
Number of HIV-infected patients who used the selected illicit drugs in their lifetime, categorized by self-reported sexual behaviors. HIV, human immunodeficiency virus; MDMA, 3,4-methyl enedioxy methamphetamine; GHB, gamma hydroxybutyrate.

The frequency of lifetime use of illicit drugs categorized by self-reported sexual behaviors is presented in Fig 1. The most commonly used drugs were cannabis/marijuana (47.7%) and methamphetamine (40%), followed by nitrite inhalants (10.8%), cocaine (9.2%), dextromethorphan (4.6%), carisoprodol (4.6%), 3,4-methylenedioxymethamphetamine (MDMA) (4.6%), synthetic cannabinoids (3.1%), and barbiturate (3.1%). The specific drugs used by five patients were not identified. Cannabis/marijuana and methamphetamine were frequently abused by both men who had sex with men (MSM) and heterosexuals, but nitrite inhalants were exclusively used by MSM.

**Fig 2:**
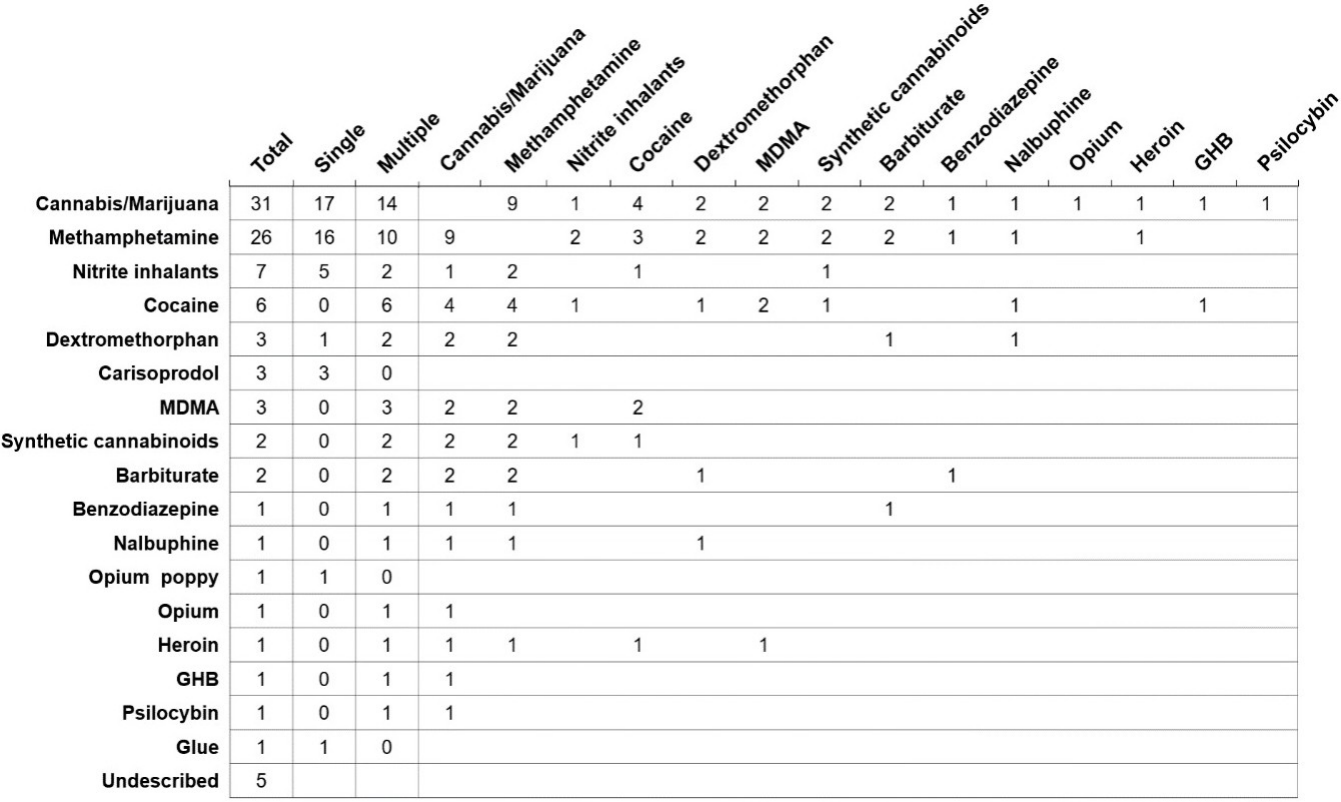
Cross-chart showing drug preferences among specific drugs in patients reported to use multiple types of drugs in their lifetime. MDMA, 3,4-methyl enedioxy methamphetamine; GHB, gamma hydroxybutyrate.

Among 65 users of illicit drugs, 16 (24.6%) reported the use of multiple types of drugs in their lifetime. Among these, 8 (50%) patients reported using two different drugs, 3 (18.8%) reported using three different drugs, and 5 (31.3%) had consumed four or more different drugs. A cross-chart showing the preferences of individuals who reported using multiple types of drugs in their lifetime is displayed in Fig 2. Cannabis/marijuana, methamphetamine, nitrite inhalants, and dextromethorphan were used by both users of single and multiple drugs, whereas carisoprodol was used by users of a single drug type, and cocaine, MDMA, synthetic cannabinoids, and barbiturate were mainly used by users of multiple drug types. Overall, 29% of cannabis/marijuana users also used methamphetamine, and 34.6% of methamphetamine users also used cannabis/marijuana. With regard to the consumption level of the drugs, 40% of the patients used the drugs experimentally, 18.5% used them occasionally, and 36.9% used them heavily.

Among the 65 patients who reported illicit drug use, only 16 (24.6%) revealed their drug use at the first visit interview. Among them, 5 (31.3%) were referred from detention centers or prisons. During follow-up, an additional 49 (75.4%) patients were identified as illicit drug users, and 5 (10.2%) of them were found to be drug users as they had been arrested for methamphetamine use.

A comparison of the characteristics of 33 users of injection drugs and 32 users of non-injection drugs is presented in Table 2. Compared with the users of non-injection drugs, those who used injection drugs were more frequently arrested for drug-related charges (48.5% vs. 3.1%, p < 0.001) and were more likely to be heavier users (66.7% vs. 6.2%, p < 0.001). The users of injection drugs reported using two or more types of drugs in their lifetime more frequently (36.4% vs. 12.5%, p = 0.003). The HCV seropositive rate was significantly higher among the users of injection drugs than among the users of non-injection drugs (50% vs. 0%). In contrast, 71.9% users of non-injection drugs reported using drugs 1–2 times in their lifetime. The users of non-injection drugs were also more likely to start using drugs overseas for the first time than the users of injection drugs (37.5% vs. 9.1%, p = 0.004), but were less likely to report their drug use in the first interview than the users of injection drugs (6.3% vs. 42.4%, p < 0.001).

**Table 2:** Drug-related characteristics of HIV-infected users of illicit drugs.

Variable	Total (n = 65)	Non-injection (n = 32)	Injection (n = 33)	P-value
Sex				0.238^b^
Male	62 (95.4)	32 (100)	30 (90.9)	
Female	3 (4.6)	0 (0)	3 (9.1)	
Age, years	39.3 ± 10.6	37.8 ± 10	40.8 ± 11.1	0.260 ^c^
Age, years				0.581^a^
≤ 30	15 (23.1)	9 (28.1)	6 (18.2)	
31–50	40 (61.5)	19 (59.4)	21 (63.6)	
> 50	10 (15.4)	4 (12.5)	6 (18.2)	
Sexual behavior				0.302 ^a^
Heterosexual	18 (27.7))	7 (21.9))	11 (33.3)	
Homosexual/bisexual	47 (72.3)	25 (78.1))	22 (66.7)	
Smoking				0.210 ^b^
Never	8 (12.3)	5 (15.6)	3 (9.1)	
Ever	54 (83.1)	27 (84.4)	27 (81.8)	
Unrecorded	3 (4.6)	0 (0)	3 (9.1)	
Disclosure of drug use at presentation to care	16 (24.6)	2 (6.2)	14 (42.4)	0.001^a^
Self-reported	11 (16.9)	2 (6.3)	9 (27.3)	
Referral from detention center or prison	5 (7.7)	0 (0)	5 (15.2)	
Number of illicit drugs used in lifetime				0.003 ^b^
Single	45 (69.2)	28 (87.5)	17 (51.5)	
Multiple	16 (24.6)	4 (12.5)	12 (36.4)	
Unrecorded	4 (6.2)	0 (0)	4 (12.1)	
Drug-related arrest or imprisonment				<0.001^b^
Never	44 (67.7)	31(96.9)	13 (39.4)	
Ever	17 (26.2)	1 (3.1)	16 (48.5)	
Unrecorded	4 (6.2)	0 (0)	4 (12.1)	
First place where they used the drug				0.004 ^b^
Domestic	46 (70.8)	20 (62.5)	26 (78.8)	
Overseas	15 (23.1)	12 (37.5)	3 (9.1)	
Unrecorded	4 (6.5)	0 (0)	4 (12.1)	
Frequency of drug use				<0.001^b^
Experimental use	26 (40.0)	23 (71.9)	3 (9.1)	
Occasional use	12 (18.5)	7 (21.9)	5 (15.2)	
Heavier use	24 (36.9)	2 (6.2)	22 (66.7)	
Unrecorded	3 (4.6)	0 (0)	3 (9.1)	
HBV seropositive	6/60 (10)	5/31(16.1)	1/29 (3.4)	0.196^b^
HCV seropositive	14/59 (23.7)	0/31 (0)	14/28 (50)	<0.001^a^

^a^ Chi-squared test.

^b^ Fisher’s exact test.

^c^ Student’s *t*-test.

Values are presented as mean ± SD, number (%).

HIV, human immunodeficiency virus; HBV, hepatitis B virus; HCV, hepatitis C virus.

**Fig 3:**
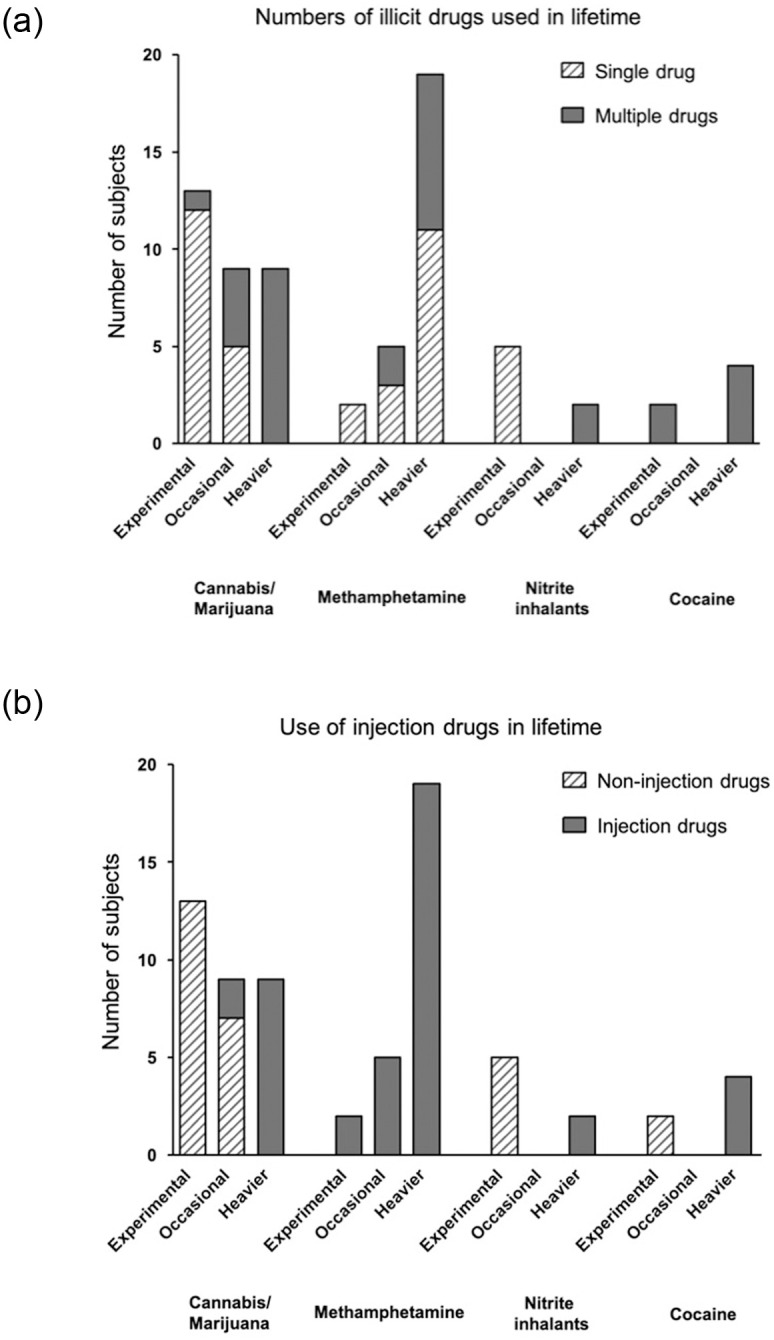
Life-time use patterns of four most frequently reported illicit drugs, categorized by drug consumption levels. A: Numbers of illicit drugs used in a lifetime, B: Use of injection drugs in a lifetime.

Fig 3 presents the drug use patterns of four most frequently reported illicit drugs, categorized by drug consumption levels. Those who reported the experimental use of cannabis/marijuana were more likely to use single drugs (92.3%) and non-injection drugs (100%). In contrast, all patients who reported heavier use of cannabis/marijuana were injection drug users, and reported using multiple types of drugs in their lifetime. Of the 19 patients who reported heavier use of methamphetamine, 42.1% reported that they had also used other types of drugs in their lifetime.

## Discussion

In this retrospective cross-sectional study, we found that approximately 5% of HIV-infected patients had used one or more illicit drugs in their lifetime, and approximately half of them used an injection drug. Although the reported prevalence of illicit drug use by patients infected with HIV can substantially vary among studies due to the differences in the definition of illicit drugs, subject characteristics, and investigated period, the prevalence observed in the present study is substantially lower than that in many other countries, where it ranges from 12% to 86% [1,13–18]. Nevertheless, this is significantly higher than the prevalence of 0.045%–0.07% in all selected subjects reported by previous studies in Korea [6,7]. There can be several reasons for this difference, but under-reporting might be an important factor. Users of illicit drugs are among the difficult-to-reach and hidden individuals, and it is difficult to obtain accurate data of such individuals [19,20]. Fear of arrest, worry of confidentiality breach, and stigma may prevent people who use illicit drugs from self-reporting. Even if confidentiality is assured, given that the use of illicit drugs is a crime, there may be cases when the use of illicit substances is either denied or under-reported [21]. As surveillance data by the KCDC are obtained by interviews between patients and public officials conducted in local community health centers immediately after diagnosis, patients may not be able to honestly report about their drug use or sexual behavior [6]. Patients in custody may not be included in the cohort study or surveillance data from the KCDC, and this could also lead to under-reporting [7,9]. In addition, because we examined lifetime drug use with repeated questioning, the estimates could be higher than those of previous studies. Nevertheless, in this study, only 24.6% of illicit drug users disclosed their drug use at initial presentation for HIV care. Furthermore, considering that 31.3% of them were referred from detention centers or prison, only 16.9% of patients initially self-reported their drug use to their healthcare providers; this shows that under-reporting is quite common in the first visit interviews. These findings indicate that additional interviews on drug use are needed to reduce under-reporting at the subsequent hospital visits. In this study, the users of non-injection drugs were less likely to report their drug use history in the first visit interview than the users of injection drugs. This finding may be partly due to a relatively high initial reporting rate of injection drug users who were referred from detention centers or prisons. In addition to the fear of stigmatization or discrimination, the users of non-injection drugs may also consider that their previous drug use was a minor event to be self-reported, considering that the majority of them reported experimental drug use [21]. This finding suggests that it may be helpful to ask more detailed questions, such as the name of the specific drug used, to reduce under-reporting than simply enquiring about the use of injection drugs.

In this study, cannabis/marijuana and methamphetamine were the most commonly reported illicit drugs. The use of cannabis/marijuana and methamphetamine was reported by 2.5% and 2.1% of all 1,267 patients infected with HIV, respectively. In the general population of Korea, based on the number of drug-related arrests, methamphetamine was reported to be the most abused drug followed by cannabis and opiates [5]. Considering that approximately 87% of the drug-related arrests recorded in our study were related to methamphetamine, our findings are in line with those for the general population of Korea [2,5,22]. Overall, 24.6% of the drug users reported using multiple types of drugs. Approximately 14% users of illicit drugs and 27.3% users of injection drugs used both cannabis/marijuana and methamphetamine in their lifetime. Cocaine, MDMA, synthetic cannabinoids, and barbiturates were used only by those who used multiple types of illicit drugs. In all, 12.9% users of cannabis/marijuana and 11.5% users of methamphetamine also used cocaine. In this study, we were not able to examine the pattern of polydrug use (taking multiple drugs at once), but these findings suggest that some users of multiple types of drugs might be polydrug users. Of note, in our study, nitrite inhalants, such as RUSH, were also commonly reported, particularly by all MSM. These products have been reported to be frequently used as recreational drugs among MSM, and they may act as a potential cofactor in the transmission of HIV [23,24]. Consistent with the findings of previous studies, all nitrite-inhalant users in our study were identified as MSM [23,24]. The percentage of MSM among the users of illicit drugs was 72.3%, which was higher than 44.21% among individuals who did not use illicit drugs.

We found some differences in the pattern of drug use between the users of injection and non-injection drugs. Compared with the users of injection drugs, the users of non-injection drugs are more likely to use a single type of substance, use it temporarily, and experience it for the first time abroad. In contrast, 36.4% users of injection drugs reported that they had used more than one class of substances in their lifetime and 66.7% were habitual users. In addition, almost half of the users of injection drugs had been arrested for drug-related charges. Among the 16 patients arrested for injection drug use, 94.1% were arrested for using methamphetamine. Half of them were incarcerated for drug offenses multiple times. Of note, five patients who did not report their drug use at initial presentation were arrested for using methamphetamine, during their follow-up period. These findings raise the concern that methamphetamine use may contribute to HIV transmission in Korean people who use injection drugs.

Coinfection with HIV and HCV is common because both infections have common routes of transmission. In our study, the prevalence of HCV coinfection in the 1,267 patients infected with HIV was estimated to be 4.9%. However, 23.7% users of illicit drugs showed evidence for coinfection with HCV, and all of them used injection drugs; therefore, the coinfection rate among the users of injection drugs was as high as 50%.

Our findings should be considered in the context of several limitations. First, this was primarily a hospital-based, descriptive, cross-sectional, prevalence study that was retrospective in nature. The collection of epidemiological information might have been limited by the retrospective nature of the study. Therefore, we cannot rule out the presence of unmeasured confounding factors. Second, this study was conducted in a single center, and a small number of patients with HIV infection were included. We included approximately 10% of the Korean population with HIV infection. Although there could be regional differences, the number of arrests for drug offenses in the southeastern region of Korea accounted for 16.8% of the total arrests in Korea in 2018 [2,25]. Considering that the population of this region accounts for 16% of the total population of Korea, our results may not reflect regional differences. Nevertheless, given that about 15% of patients were referred from the detention centers or prisons in our study, there may be a sampling bias depending on their prisoner placement or the hospital referral process, and these results can be generalized to other regions of the country with caution. Third, we evaluated drug use patterns across a broad time frame, which can be beneficial as it can reduce under-reporting and assess the changes in drug use patterns over time. Nevertheless, the interview was conducted as a part of the routine clinical setting and was not anonymous. Furthermore, we relied on self-reporting, and socially stigmatized behaviors may have therefore been underreported. Further studies such as computer-assisted approach or actual drug testing are needed for more accurate estimation. We also could not link the episodes of drug use directly to the sexual risk behavior. Fourth, non-ethnic Koreans were excluded from this study because 72.1% (31/43) of them were lost to follow-up or returned to their countries after temporary visits for overseas employment (16), marriage (11), detention (2), and visiting hospital (1) and relatives (1), and there were also some limitations in obtaining information due to language barrier or concerns about visa suspension.

## Conclusions

To our knowledge, this is the first study to examine in-depth use of illicit drugs by people with HIV infection in Korea. The prevalence of illicit drug use among people with HIV infection was higher than that reported in previous studies in Korea [6,7]. In this study, under-reporting was also common until a relationship of trust was established between the doctor and patient. Clinicians should pay more attention toward the collection of information about illicit drug use at the first visit interview, and repeated questioning on drug use during follow-up would help identify more drug users. Cannabis/marijuana and methamphetamine were the most commonly reported illicit drugs. The prevalence of illicit drug use in people with HIV infection was higher in MSM than in heterosexuals. The users of injection drugs are more likely to be habitual users, use more than one class of substances, and are more likely to be arrested on drug-related charges. Our results indicate that further studies on the prevalence of illicit drug use in people with HIV infection in Korea based on more accurate measurements are urgently needed. Our findings also underscore the need for further research on the relationship between illicit drug use and sexual risk behavior. Changing patterns of drug use should be monitored, and education and intervention programs on HIV prevention are also needed.
